# Emergency Ventral Hernia Management in Older Adults: A Retrospective Cohort Study and Structured Review of the Literature

**DOI:** 10.3390/geriatrics11020036

**Published:** 2026-03-27

**Authors:** Ivan Tomasi, Jeremy Samuel, Eimante Raupelyte, Antonia Elizabeth Loizou, Angela Wang Yihui, Lilian Chioma Ujunwa Nwosu, Sneha Mehrotra, Mariia Druziagina, Kenneth Wing Ngai Law, Magda Sbai

**Affiliations:** 1 Department of General Surgery, Guy’s and St Thomas’ NHS Foundation Trust, London, SE1 9RT, UK; 2 School of Medicine, King’s College London, London WC2R 2LS, UK; 3 Perioperative Medicine for Older People Undergoing Surgery (POPS) Service, Guy’s and St Thomas’ NHS Foundation Trust, London SE1 9RT, UK

**Keywords:** emergency ventral hernia, older adults, frailty, emergency general surgery, postoperative outcomes

## Abstract

**Background/Objectives**: Older adults frequently present with emergency ventral hernias, a situation that carries significant physiological risks and often requires challenging clinical decisions. Despite the prevalence of these cases, there is a lack of robust evidence to inform emergency care in this demographic, as most existing research centres on short-term mortality rates and operative variables. Key aspects such as the impact of frailty and the course of recovery following surgery are insufficiently addressed in the literature. This study aimed to describe management strategies, frailty burden and postoperative outcomes in older adults presenting with emergency ventral hernias. **Methods**: This study retrospectively examined patients aged 65 and older who were admitted to a UK tertiary centre with emergency ventral hernias from February 2016 to July 2024. Data, including patient demographics, comorbid conditions, frailty status (as measured by the Clinical Frailty Scale), management approach, healthcare resource use, and clinical outcomes, were analysed descriptively. Additionally, a structured literature review was conducted in accordance with PRISMA guidelines to identify research on emergency ventral hernia treatment outcomes in adults aged 60 years and older. **Results**: A total of 67 patients met the inclusion criteria for the cohort. High rates of frailty and multiple coexisting health conditions were observed. While surgical intervention was the predominant management strategy, a subset of patients received conservative or palliative care. Greater degrees of frailty correlated with longer hospital stays and an increased need for critical care, even though six-month mortality remained comparatively low. Traditional risk assessment tools tended to overpredict mortality risk and failed to reflect the true postoperative burden or the recovery process. The systematic review yielded 7 studies, most of which documented mortality and complication rates, but few addressed frailty or provided detailed postoperative recovery data. **Conclusions**: The management of emergency ventral hernias in older adults is highly variable, with a significant postoperative impact that extends beyond mortality statistics. Assessing frailty appears to provide additional information that may support clinical decision-making and help anticipate recovery after surgery. Integrating frailty evaluation into emergency hernia care could enhance multidisciplinary collaboration and help ensure that treatment plans are better tailored to patient vulnerability and individual care goals.

## 1. Introduction

Emergency ventral hernias are a frequent and important challenge in general surgical practice globally. These hernias—including primary, incisional, paraumbilical, and parastomal types—are estimated to affect between 10% and 20% of all adults over their lifetimes, with the likelihood increasing with age and a history of abdominal surgery [1,2]. Large-scale data indicate that about 8–10% of all ventral hernia repairs are undertaken emergently, most often prompted by incarceration or strangulation [3]. For individuals with untreated ventral hernias, the annual risk of developing acute complications such as incarceration or strangulation remains relatively low but is still clinically relevant. Emergency surgery is most required for patients with irreducible hernias, clinical evidence of strangulation or obstruction, or severe, unmanageable pain, and such procedures are associated with greater morbidity and resource use compared to elective operations [4].

Older individuals make up a significant proportion of patients requiring emergency surgery, largely because of ageing, physiological resilience, the presence of multiple health conditions, and declining functional abilities. In most surgical research and clinical settings, ‘older adults’ are defined as those aged 65 and above, although some studies adopt a threshold of 60 years for epidemiological purposes [5,6,7,8,9,10,11]. Nonetheless, using age as the sole marker of vulnerability can be misleading. Frailty—a condition marked by increased sensitivity to stressors and driven by cumulative deterioration across several bodily systems—has emerged as a more accurate predictor of surgical risk than age by itself. Tools such as the Clinical Frailty Scale (CFS) provide a structured way to assess baseline frailty and have been shown to be associated with outcomes after acute surgical interventions [12].

Although frailty is widely acknowledged as a crucial factor in surgical risk for older adults, most research on emergency ventral hernia management remains focused on short-term mortality and intraoperative variables, with little emphasis on frailty or patients’ functional recovery [5,6,7,8,9,10,11]. Standard risk assessment models—such as the American Society of Anaesthesiologists (ASA) classification, Portsmouth Physiological and Operative Severity Score for the enumeration of Mortality (P-POSSUM), and the National Emergency Laparotomy Audit (NELA)—are routinely used in emergency settings, but these tools primarily estimate mortality risk and rarely consider frailty or the broader postoperative experience [13]. As a result, the connection between frailty, clinical choices, and recovery patterns in older patients facing emergency ventral hernia surgery is not well explored.

This study set out to characterise the practical management of emergency ventral hernias among older adults in a UK hospital setting, with a specific focus on the influence of frailty and recovery patterns after surgery. Additional goals included assessing healthcare resource use and clinical outcomes beyond mortality and situating these findings within the context of existing research through a structured literature review.

## 2. Materials and Methods

We performed a retrospective observational cohort analysis involving older adults who were admitted with emergency ventral hernias. Alongside this, we conducted a structured literature review to provide a broader context for the cohort data.

## 3. Results

### 3.1. Setting

The research took place at Guy’s and St Thomas’ NHS Foundation Trust, a major tertiary hospital in London, UK, that offers emergency general surgery. Eligible patients were identified from emergency surgical admissions occurring between February 2016 and July 2024. Information on follow-up, including survival status, was collected from electronic medical records for up to 6 months after the initial admission. At our institution, the POPS service runs Monday to Friday, in hours. Older high-risk emergency surgical patients are reviewed in a multidisciplinary meeting with surgeons, geriatricians, and anaesthetists prior to any operative decision, where possible.

### 3.2. Participants

Inclusion criteria comprised adults aged 65 or older who were admitted for emergency evaluation of a ventral hernia. Emergency ventral hernias were defined as ventral, incisional, paraumbilical, or parastomal hernias presenting acutely and requiring surgical intervention after assessment by a surgical consultant. Patients admitted solely for elective hernia repair were excluded from the study.

Management approaches during the initial hospitalisation were classified as either operative or non-operative. Operative interventions encompassed emergency repair procedures, which could involve bowel resection or the use of mesh. Non-operative strategies consisted of conservative measures, symptom management, and discharge without surgery.

### 3.3. Variables

Baseline characteristics recorded included patient age, sex, body mass index, comorbidities, history of abdominal surgery, and hernia type. Frailty was measured using the Clinical Frailty Scale (CFS) at hospital admission.

Assessment of physiological risk included the ASA physical status classification and, when available, P-POSSUM mortality predictions. NELA Mortality risk was also recorded if documented during routine clinical practice.

The involvement of the Perioperative Medicine for Older People Undergoing Surgery (POPS) team was documented in accordance with routine clinical practice. When the POPS team was involved, geriatric input was provided as part of routine clinical care, including assessment of comorbidities, functional status, cognition, medication review, and perioperative optimisation. However, a formal, standardised, multidimensional, comprehensive geriatric assessment was not uniformly performed for all patients. Primary outcomes focused on the postoperative course and hospital resource use, specifically length of stay and the need for critical care. Secondary endpoints included the type of management strategy employed, the occurrence of postoperative complications, and mortality within six months.

### 3.4. Data Sources and Measurement

Data collection involved reviewing electronic patient records, surgical and anaesthetic documentation, and institutional audit databases. Information on frailty, physiological risk, POPS team involvement, and outcomes was recorded as noted in routine practice. Missing data were not imputed.

### 3.5. Bias

Given the retrospective observational design, potential sources of bias included selection bias and confounding by indication—especially regarding decisions about operative versus nonoperative care and referral to the POPS team. Analyses were descriptive, and no attempts were made to establish causal relationships.

### 3.6. Study Size

Study size was determined by the total number of patients who met the inclusion criteria during the study timeframe. No formal sample-size estimation was conducted.

### 3.7. Statistical Analysis

Descriptive statistics were generated using Microsoft Excel for Microsoft 365 (Microsoft Corporation, Redmond, WA, USA).

Continuous variables were summarised as medians and interquartile ranges, and categorical variables as counts and percentages. Outcomes were reported for the overall cohort and, where appropriate, stratified by frailty category. No multivariable statistical modelling was performed.

Exploratory non-parametric analyses were performed to compare operative versus non-operative management and frail versus non-frail patients. Continuous variables were compared using the Mann–Whitney U test, and categorical variables using Fisher’s exact test. Given the small sample size and retrospective design, these analyses were considered exploratory and hypothesis-generating.

A structured literature review was undertaken concurrently with the cohort study, following the Preferred Reporting Items for Systematic Reviews and Meta-Analyses (PRISMA) guidelines [14]. The objective was to identify studies reporting outcomes of emergency ventral hernia cases in older adult populations.

A structured literature search was conducted in MEDLINE, Embase, and the Cochrane Library, with results reported from database inception through July 2024. The search strategy incorporated both Medical Subject Headings (MeSH) and free-text keywords related to ventral hernia, emergency presentation, and older age. The complete list of search terms and Boolean operators is detailed in Supplementary Table S1.

**Figure 1:**
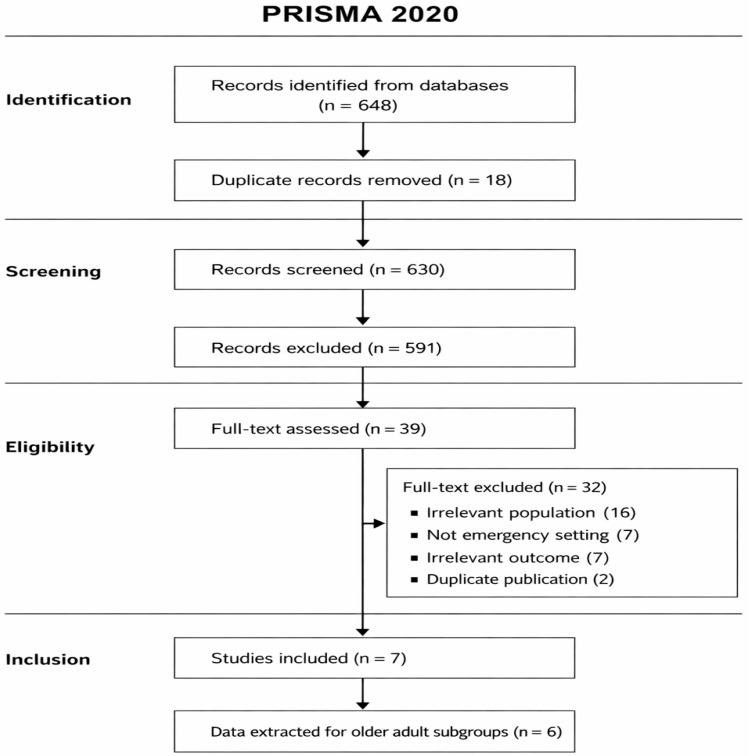
PRISMA flow diagram illustrating study selection for the structured review of the literature.

Screening of studies was initially performed on titles and abstracts, followed by a review of full texts. Inclusion criteria encompassed studies reporting on emergency ventral hernia outcomes in adults aged 60 years or older, or those with extractable data for this age group. Studies focused exclusively on elective repairs or lacking relevant outcomes were excluded. The selection process is illustrated in the PRISMA flow diagram (Figure 1).

Reporting of this research follows the Strengthening the Reporting of Observational Studies in Epidemiology (STROBE) statement for cohort studies [15].

## 4. Expanded Results

Sixty-seven older adults (median age 73 [IQR 67.5–79.0]; 59.7% female) were admitted with emergency ventral hernias between February 2016 and July 2024 and included in the cohort. All patients fulfilled the predefined inclusion criteria, with no exclusions after eligibility assessment. Complete follow-up, including six-month mortality data, was obtained for 100% of the cohort.

There was a high prevalence of comorbidities: 73.1% had ≥2 chronic conditions, and 80.6% (54/67) had a history of prior abdominal surgery. Frailty and vulnerability were common, with 52.2% of patients classified as vulnerable or frail (Clinical Frailty Scale ≥ 4), including 25.4% meeting criteria for frailty (CFS ≥ 5). Baseline demographic and clinical characteristics are detailed in Table 1.

The Perioperative Medicine for Older People Undergoing Surgery (POPS) team was involved in 77.6% of admissions, providing geriatric input as part of multidisciplinary perioperative care to guide perioperative and postoperative management. POPS involvement was more common in patients with higher levels of frailty and comorbidity.

Most patients (79.1%) underwent operative management during the index admission, while 20.9% were managed non-operatively. Non-operative management was more frequent among patients with advanced frailty, high physiological risk, or where goals of care prioritised conservative or palliative approaches. Details of management strategies are provided in Table 2. The median hospital stay was 9 days [IQR 5–17], with longer stays observed among frail patients. Admission to critical care was required in 26.9% of cases, occurring exclusively among those who underwent surgery and more frequently in frail individuals.

Surgical field contamination varied across hernia subtypes, with a significant proportion of cases classified as clean–contaminated or contaminated, and a smaller number as dirty. This was particularly evident in parastomal and complex ventral hernias. These findings confirm that not all procedures were performed in clean conditions and support the selective use of mesh based on intraoperative contamination risk, as summarised in Table 3.

Physiological risk stratification data were not always available: ASA grade was recorded in 17.9% of patients, P-POSSUM estimates in 38.8%, and NELA-predicted mortality was recorded in 45 of 67 patients (67.2%). Across the cohort, predicted mortality frequently exceeded observed outcomes, with median predicted mortality of 7.1% (NELA) and 9.1% (P-POSSUM) compared with an observed six-month mortality of 6.0% (4/67 patients). Comparative data are summarised in Table 4.

Postoperative complications occurred in 32.1% (17/53) of patients who underwent surgery, with most being low- or moderate-grade (Clavien-Dindo I–II). Major complications (Clavien-Dindo ≥ III) were observed in 7.7%. Postoperative mortality was 4.5%. Among operative patients with available follow-up, hernia recurrence within 12 months occurred in 5 patients (9.4%). Complications and long-term outcomes are summarised in Table 5.

Exploratory analyses were performed to support the descriptive findings. Patients managed operatively had a significantly longer length of stay than those managed non-operatively (Mann–Whitney U test, *p* = 0.0017) and were more likely to require critical care admission (Fisher’s exact test, *p* = 0.031). Six-month mortality did not differ significantly between the two groups (*p* = 0.247).

Frail patients had a significantly longer length of stay than non-frail patients (*p* = 0.0007). Among operative patients, frailty was also associated with a longer postoperative length of stay (*p* = 0.0045). Differences in critical care admission, postoperative complications, and six-month mortality did not reach statistical significance, likely reflecting the limited sample size. Frailty-stratified outcomes are summarised in Table 5. Physiological risk stratification data and comparisons between predicted and observed mortality are summarised in Table 6. Characteristics of the included studies are summarised in Table 7.

Operative management was offered across all frailty categories (70.6% in frail patients vs. 81.3% in non-frail patients). However, advanced frailty was associated with a marked increase in postoperative burden, including a longer median length of hospital stay (18 vs. 8 days), higher rates of critical care admission (52.9% vs. 18.8%), and more complex recovery trajectories. Mortality rates did not adequately reflect these gradients in postoperative burden.

## 5. Discussion

This retrospective cohort study of 67 older adults presenting with emergency ventral hernias found that frailty was prevalent (25.4% with CFS ≥ 5) and had a significant impact on postoperative outcomes and resource use. Operative management was performed in the majority (79.1%), but patients with higher frailty experienced a longer median length of hospital stay (18 vs. 8 days), higher rates of critical care admission (52.9% vs. 18.8%), and more complex recoveries, even though six-month mortality remained low at 6.0%. These findings suggest that the overall postoperative burden and the pattern of recovery may tell us more about how patients truly fare than mortality figures alone. An important point to keep in mind when reading our results is that geriatric care in this study was not delivered through a formal, standardised assessment pathway, but rather as part of routine clinical practice. Although multidisciplinary involvement was common, the lack of a consistent and structured geriatric evaluation makes it difficult to pinpoint which specific aspects of geriatric care had the greatest impact on outcomes.

The structured review identified seven studies that reported outcomes for emergency ventral hernia management in older adults or in extractable older subgroups. Across these studies, reported mortality ranged from 3.1% to 9.5%, and morbidity rates were generally high [5,6,7,8,9,10,11]. However, most studies focused on short-term mortality and operative variables, with little detail on postoperative trajectory, length of stay, or use of critical care. Notably, none of the studies formally included frailty assessment or comprehensive geriatric evaluation.

Our findings align with the published literature on mortality, which shows low mortality in both our cohort and existing studies, despite the emergency nature of these cases [5,6,7,8,9,10,11]. By incorporating frailty assessment and examining postoperative trajectories, our study offers new perspectives on the burden of care for older adults. Specifically, we demonstrate that frail patients endure longer hospital stays and require more intensive postoperative support, highlighting an evidence gap where outcomes beyond mortality are often overlooked.

Most prior studies have defined older adults by chronological age cut-offs (typically ≥60 or ≥65 years) [6,7,8,9,10,11]. Our data reinforce the notion that age alone is insufficient to capture surgical risk. Instead, frailty—a marker of diminished physiological reserve and increased baseline vulnerability—was more closely associated with differences in postoperative course. Assessing frailty appears to provide additional information that may support clinical decision-making and help anticipate recovery after surgery. This finding aligns with broader surgical literature linking frailty to worse outcomes, yet it remains underexplored in the context of emergency ventral hernia management.

Commonly used risk stratification tools—such as ASA, P-POSSUM, and NELA—were documented for most of our patients and are well-documented in the literature [5,6,7,8,11]. These models primarily estimate short-term mortality and help guide care escalation. In our study, predicted mortality from these tools was consistently higher than observed six-month mortality (predicted median: 7.1% vs. observed: 6.0%), illustrating the limitations of relying on mortality-focused models in frail, older populations. While these tools serve important roles in perioperative management, they do not address postoperative burden, recovery potential, or functional vulnerability, which are especially pertinent for older adults.

POPS team involvement was recorded in 77.6% of cases in our cohort, reflecting current emergency surgical practice in the UK. Although we did not separately analyse the impact of POPS due to potential confounding, its routine integration highlights the value of multidisciplinary care for older surgical patients. The lack of geriatric input in published studies further underscores the novelty and clinical relevance of frailty-aware pathways in this setting [5,6,7,8,9,10,11]. Another limitation worth noting is that the POPS service at our institution currently operates only during weekday working hours. While this mirrors the reality of how such services operate in many hospitals in the UK, it means that geriatric input may not always be available when urgent decisions need to be made. Given how central multidisciplinary collaboration appeared to be in shaping outcomes, extending service availability could make a real difference in emergency settings.

**Figure 2:**
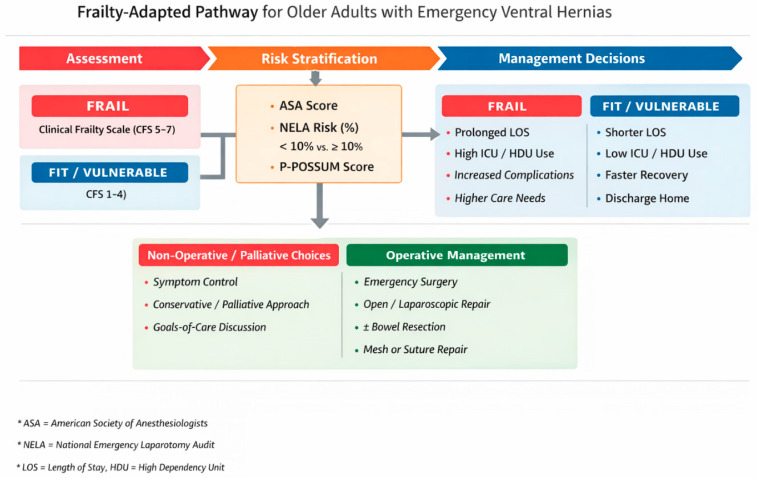
Proposed frailty-adapted pathway for the management of older adults presenting with emergency ventral hernias.

Overall, our results support the potential value of a frailty-informed approach to emergency surgical decision-making in older adults with ventral hernias. Focusing solely on mortality underestimates the complexity and true impact of emergency surgery in this group. Incorporating frailty assessment alongside physiological risk models can facilitate shared decision-making, improve perioperative planning, and better align care intensity with patient vulnerability and preferences. The frailty-adapted pathway in Figure 2 illustrates how such an approach can be operationalised in daily practice.

Strengths of this study include comprehensive clinical data, systematic assessment of frailty, and integration with a structured literature review. This study has several limitations. First, its retrospective design introduces potential selection bias, missing data, and unmeasured confounding. Second, this was a single-centre study conducted within an existing weekday POPS service model, which may limit generalizability. Third, although geriatric input was frequently provided, a standardised, comprehensive, multidimensional geriatric assessment was not consistently performed or documented. Therefore, the findings should be interpreted as reflecting geriatric co-management within a pragmatic service structure, rather than the effect of a formalised CGA-based intervention. The literature review was also constrained by heterogeneity in study designs, reporting, and age definitions across included studies, which precluded a formal meta-analysis.

Future research should prioritise prospective, multicentre studies that focus on frailty-informed outcomes in emergency ventral hernia management, including functional recovery, discharge destination, and patient-reported outcomes. Harmonising outcome definitions beyond mortality would enable better comparison across studies. Further evaluation of integrated geriatric and perioperative medicine approaches in emergency surgical populations is also warranted.

## Figures and Tables

**Table 1:** Baseline characteristics of older adults admitted with emergency ventral hernias. Data are presented as *n* (%) unless otherwise stated; continuous variables are median (interquartile range).

Characteristic	Value
Number of patients	67
Age, years	73 (IQR 67.5–79.0)
Female sex	40 (59.7%)
BMI, kg/m^2^	31.4 (IQR 25.7–37.3); missing 10
Previous abdominal surgery	54 (80.6%)
Clinical Frailty Scale (CFS), median (IQR)	4 (3–5); missing 2
Fit (CFS 1–3)	30 (44.8%)
Vulnerable (CFS 4)	18 (26.9%)
Frail (CFS 5–7)	17 (25.4%)
POPS involvement documented	52 (77.6%)
Comorbidity	*n* (%)
Cardiovascular disease	50 (74.6%)
Diabetes mellitus	21 (31.3%)
Respiratory disease	25 (37.3%)
Renal disease	23 (34.3%)
Liver disease	4 (6.0%)
History of malignancy	29 (43.3%)
Anticoagulation/antiplatelet therapy	9 (13.4%)
VH Subtype	*n* (%)
Incisional hernia	31 (46.3%)
Parastomal hernia	7 (10.4%)
Umbilical hernia	6 (9.0%)
Epigastric hernia	6 (9.0%)
Paraumbilical hernia	3 (4.5%)
Spigelian hernia	1 (1.5%)
Suprapubic hernia	1 (1.5%)
Other ventral/complex	12 (17.9%)
VH Status	*n* (%)
Reducible	8 (11.9%)
Irreducible	32 (47.8%)
Incarcerated	33 (49.3%)
Strangulated	11 (16.4%)
ASA score recorded	12 (17.9%)
—ASA II (of recorded)	4 (33.3%)
—ASA III (of recorded)	7 (58.3%)
—ASA IV (of recorded)	1 (8.3%)
NELA predicted mortality recorded	45 (67.2%)
—NELA predicted mortality, %	7.1 (IQR 3.8–12.5)
P-POSSUM mortality recorded	26 (38.8%)
—P-POSSUM predicted mortality, %	9.1 (IQR 4.1–17.7)

**Table 2:** Management strategies and operative details in older adults presenting with emergency ventral hernias.

Initial Management Strategy	
Management during index admission	*n* (%)
Operative management	53 (79.1%)
Non-operative management	14 (20.9%)
Indication for surgery	*n* (%)
Incarceration without strangulation	24 (45.3%)
Strangulation	11 (20.8%)
Irreducible hernia with pain/obstruction	13 (24.5%)
Other/mixed indications	5 (9.4%)
Operative approach	n (%)
Open repair	49 (92.5%)
Laparoscopic/minimally invasive	4 (7.5%)
Bowel involvement and resection	*n* (%)
Bowel involvement documented	17 (32.1%)
Bowel resection performed	9 (17.0%)
Primary anastomosis	7 (13.2%)
Stoma formation	2 (3.8%)
Mesh position	*n* (%)
Onlay/sublay/underlay	12 (54.5%)
Intraperitoneal	6 (27.3%)
Not specified	4 (18.2%)
Anaesthetic and perioperative context	
Variable	*n* (%)
General anaesthesia	51 (96.2%)
Consultant surgeon present	53 (100%)
Consultant anaesthetist present	41 (77.4%)
POPS involvement pre-/peri-operatively	52 (77.6%)
Non operative Strategy	*n* (%)
Conservative management and discharge	8 (57.1%)
Symptom control with inpatient observation	4 (28.6%)
Palliative/goals-of-care-led approach	2 (14.3%)

**Table 3:** Mesh use according to hernia subtype and surgical field contamination in the operative cohort.

Hernia Type	Total (*n*)	Mesh Used (*n*, %)	Mesh Type (Synthetic/Biological/Composite)	No Mesh (*n*)	Clean (*n*)	Contaminated (*n*)	Dirty (*n*)
Incisional	27	12 (44.4%)	3/5/0	15	18	7	0
Parastomal	8	0 (0%)	0/0/0	8	5	1	1
Primary ventral (umbilical/epigastric)	16	2 (12.5%)	2/0/0	14	7	4	0
Other/complex hernia	17	2 (11.8%)	1/1/0	15	8	1	8

Note: “Contaminated” includes clean-contaminated and contaminated cases. Some patients presented with multiple hernia types; therefore, totals may exceed the number of operative cases.

**Table 4:** Postoperative outcomes, resource utilization, and clinical trajectory following emergency ventral hernia admission.

Variable	Value		
Length of stay, days (overall)	9 (IQR 5–17)		
Length of stay, operative cohort (*n* = 53)	11 (IQR 6–20)		
Length of stay, non-operative cohort (*n* = 14)	4 (IQR 2–7)		
Critical care utilisation			
Variable	*n* (%)		
Admission to ICU/HDU (overall)	18 (26.9%)		
ICU/HDU admission—operative cohort	18/53 (34.0%)		
ICU/HDU admission—non-operative cohort	0/14 (0%)		
Duration of ICU/HDU stay, days	2 (IQR 1–5)		
Postoperative complications			
*(operative cohort, n = 53)*			
Complication category	*n* (%)		
Any postoperative complication	17 (32.1%)		
Surgical site infection	9 (17.0%)		
Respiratory complications	7 (13.2%)		
Cardiovascular complications	4 (7.5%)		
Delirium	6 (11.3%)		
Acute kidney injury	5 (9.4%)		
Return to theatre	4 (7.5%)		
Severity of complications (Clavien–Dindo)			
*(operative cohort, n = 53)*			
Grade	*n* (%)		
Grade I–II	11 (20.8%)		
Grade III	4 (7.5%)		
Grade IV	2 (3.8%)		
Grade V (death)	2 (3.8%)		
Mortality			
Time point	*n* (%)		
In-hospital mortality	1 (1.5%)		
30-day mortality	2 (3.0%)		
6-month mortality	4 (6.0%)		
Discharge destination			
Destination	*n* (%)		
Home (independent or with support)	46 (68.7%)		
Rehabilitation/step-down facility	13 (19.4%)		
Care home	4 (6.0%)		
Died during index admission	1 (1.5%)		
Hernia recurrence and re-presentation			
Variable	*n* (%)		
Hernia recurrence within 12 months *	5 (9.4%)		
Emergency re-presentation within 30 days	6 (9.0%)		
Frailty-stratified postoperative trajectory			
Outcome	Fit (CFS 1–3)	Vulnerable (CFS 4)	Frail (CFS ≥ 5)
Median LOS, days	6	10	18
ICU/HDU admission	13.3%	27.8%	52.9%
Any complication	20.0%	27.8%	52.9%
6-month mortality	3.3%	5.6%	11.8%

Note: * Recurrence assessed from available follow-up documentation.

**Table 5:** Frailty-stratified management strategies, postoperative trajectory, and clinical outcomes according to Clinical Frailty Scale category.

Frailty Category	Definition	*n* (%)	
Fit	CFS 1–3	30 (44.8%)	
Vulnerable	CFS 4	18 (26.9%)	
Frail	CFS ≥ 5	17 (25.4%)	
Total		65 *	
Management	Fit	Vulnerable	Frail
Operative management	25 (83.3%)	14 (77.8%)	12 (70.6%)
Non-operative management	5 (16.7%)	4 (22.2%)	5 (29.4%)
Palliative/goals-of-care-led	0 (0%)	1 (5.6%)	2 (11.8%)
Postoperative trajectory and resource utilisation			
Length of stay, days (median)	6	10	18
ICU/HDU admission	4 (13.3%)	5 (27.8%)	9 (52.9%)
ICU/HDU stay ≥ 48 h	1 (3.3%)	2 (11.1%)	6 (35.3%)
Postoperative complications (operative cohort)			
Any complication	6 (20.0%)	5 (27.8%)	9 (52.9%)
Clavien–Dindo ≥ III	1 (3.3%)	2 (11.1%)	3 (17.6%)
Postoperative delirium	1 (3.3%)	2 (11.1%)	3 (17.6%)
Mortality and discharge outcomes			
In-hospital mortality	0 (0%)	0 (0%)	1 (5.9%)
30-day mortality	1 (3.3%)	0 (0%)	1 (5.9%)
6-month mortality	1 (3.3%)	1 (5.6%)	2 (11.8%)
Discharge home	24 (80.0%)	12 (66.7%)	7 (41.2%)
Discharge to rehabilitation/care facility	5 (16.7%)	5 (27.8%)	7 (41.2%)

* Frailty score missing in 2 patients.

**Table 6:** Physiological risk stratification and comparison between predicted and observed mortality outcomes.

Risk Stratification Tool	Available, *n* (%)	
ASA physical status	12/67 (17.9%)	
P-POSSUM predicted mortality	26/67 (38.8%)	
NELA predicted mortality	45/67 (67.2%)	
Risk tool	Predicted mortality (%)	
P-POSSUM (*n* = 26)	9.1 (IQR 4.1–17.7)	
NELA (*n* = 45)	7.1 (IQR 3.8–12.5)	
Observed mortality	*n* (%)	
In-hospital mortality	1/67 (1.5%)	
30-day mortality	2/67 (3.0%)	
6-month mortality	4/67 (6.0%)	
Predicted vs. observed mortality (descriptive comparison)	Value	
Median predicted mortality (P-POSSUM)	9.1%	
Median predicted mortality (NELA)	7.1%	
Observed 30-day mortality	3.0%	
Observed 6-month mortality	6.0%	
High-risk thresholds and clinical context	*n* (%)	
NELA predicted mortality ≥ 10%	14/45 (31.1%)	
Patients requiring ICU/HDU admission	18/67 (26.9%)	
Consultant surgeon present (operative cases)	53/53 (100%)	
Consultant anaesthetist present	41/53 (77.4%)	
Outcome: Relationship between risk scores and postoperative trajectory	Low–moderate predicted risk	High predicted risk *
Median length of stay, days	Shorter	Prolonged
ICU/HDU admission	Less frequent	More frequent
Mortality	Low	Remained low
Postoperative burden	Moderate	Substantial

Note: * High predicted risk defined pragmatically as NELA predicted mortality ≥ 10%.

**Table 7:** Summary of studies included in the structured literature review on emergency ventral hernia outcomes in older adults.

Study (Author, Year)	Country	Study Design	Population/Age Definition	Emergency-Only Cohort	Main Outcomes Reported	Key Limitations
Spaniolas et al., 2014 [5]	USA	Retrospective database study	≥80 years	Mixed	Mortality, morbidity	Limited reporting of LOS and ICU use
Özkan et al., 2012 [10]	Turkey	Retrospective single-centre	Mean age ≥ 60	Yes	In-hospital mortality, complications	No frailty assessment; limited outcome detail
Huckaby et al., 2020 [9]	USA	Retrospective multi-centre	Mean age ~62	Mixed (acute hernias)	Mortality, ICU admission, LOS	No postoperative trajectory outcomes
Surek et al., 2020 [6]	Germany	Retrospective single-centre	Mean age ≥ 60	Yes	Morbidity, mortality	Limited reporting of LOS and ICU use
Proaño-Zamudio et al., 2022 [7]	Mexico	Retrospective database study	≥65 years	Yes	In-hospital mortality, LOS	Small cohort; no frailty or functional data
Patel et al., 2022 [8]	USA	Retrospective database study	≥60 years	Yes	Mortality, complications, LOS	Administrative data; no clinical granularity
Thalpati et al., 2025 [11]	India	Retrospective single-centre	≥60 years	Yes	In-hospital mortality, LOS	Limited outcome reporting

## Data Availability

The datasets generated and/or analyzed during the current study are not publicly available due to patient confidentiality and institutional data governance restrictions, but are available from the corresponding author on reasonable request.

## Supplementary Materials

The following supporting information can be downloaded at: https://www.mdpi.com/article/10.3390/geriatrics11020036/s1, Table S1: Search Strategy and Boolean Terms Used for the Structured Literature Review.
