# Supplementary material for: Emergency Ventral Hernia Management in Older Adults: A Retrospective Cohort Study and Structured Review of the Literature
Source: Geriatrics (Basel). 2026 Mar 27;11(2):36. doi: 10.3390/geriatrics11020036 (PMC13116691; doi:10.3390/geriatrics11020036)
Supplement: Supplementary file 1 [file geriatrics-11-00036-s001.zip › geriatrics-4211704-supplementary.pdf]

Table S1. Search Strategy and Boolean Terms Used for the Structured Literature Review.

Database	Search strategy
**PubMed (MEDLINE)**	("ventral hernia"[MeSH Terms] OR "ventral hernia"[Title/Abstract] OR "incisional hernia"[Title/Abstract] OR "abdominal wall hernia"[Title/Abstract] OR "paraumbilical hernia"[Title/Abstract] OR "umbilical hernia"[Title/Abstract] OR "parastomal hernia"[Title/Abstract]) AND ("emergency"[Title/Abstract] OR "acute"[Title/Abstract] OR "incarcerated"[Title/Abstract] OR "strangulated"[Title/Abstract] OR "complicated"[Title/Abstract]) AND ("elderly"[Title/Abstract] OR "older adults"[Title/Abstract] OR "aged"[MeSH Terms] OR "aged 65"[Title/Abstract] OR "≥60 years"[Title/Abstract] OR "geriatric"[Title/Abstract])
**Embase**	('ventral hernia'/exp OR 'ventral hernia':ti,ab OR 'incisional hernia':ti,ab OR 'abdominal wall hernia':ti,ab OR 'paraumbilical hernia':ti,ab OR 'umbilical hernia':ti,ab OR 'parastomal hernia':ti,ab) AND ('emergency':ti,ab OR 'acute':ti,ab OR 'incarcerated':ti,ab OR 'strangulated':ti,ab OR 'complicated':ti,ab) AND ('elderly'/exp OR 'aged'/exp OR 'older adult':ti,ab OR 'elderly':ti,ab OR '≥60 years':ti,ab OR 'geriatric':ti,ab)
**Cochrane Library (CENTRAL)**	("ventral hernia" OR "incisional hernia" OR "abdominal wall hernia" OR "paraumbilical hernia" OR "umbilical hernia" OR "parastomal hernia") AND ("emergency" OR "acute" OR "incarcerated" OR "strangulated" OR "complicated") AND ("elderly" OR "older adults" OR "aged" OR "≥60 years" OR "geriatric")
